# Collagen catabolism through Coll2-1 and Coll2-1NO_2_ and myeloperoxidase activity in marathon runners

**DOI:** 10.1186/2193-1801-2-92

**Published:** 2013-03-08

**Authors:** Yves Henrotin, Alain Labasse, Thierry Franck, Alain Bosseloir, Thierry Bury, Michelle Deberg

**Affiliations:** 1 Bone and Cartilage Research Unit, University of Liège, CHU Sart Tilman, Liège, Belgium; 2 Artialis SA, Avenue de l’Hôpital, GIGA Tower +3, B34, Sart Tilman, Liège, Belgium; 3 Centre for Oxygen Research and Development, Institute of Chemistry, University of Liège, Liège, Belgium; 4 Zentech SA, Liège Science Park, Angleur, Belgium; 5 Department of Sport Physiology, University of Liège, Liège, Belgium

**Keywords:** Cartilage, Endurance exercise, Type II collagen, Biomarkers

## Abstract

To determine the influence of marathon on the serum levels of two markers of cartilage degradation, Coll2-1 and its nitrated form, Coll2-1NO_2_, and of a marker of neutrophils activation, the myeloperoxidase (MPO).

Coll2-1, Coll2-1NO_2_, total and active MPO were measured in 98 marathon runners without joint pain and with an average age of 47 years. Sera were taken at rest right before the departure and within 30 min after the marathon. The subjects were submitted to a questionnaire concerning their physical activity and their life style.

The levels of Coll2-1, Coll2-1NO_2_ and active MPO were not affected by age, body mass index, sex or performance. The levels of total MPO were higher in female than in male (p < 0.05), but were not affected by the other parameters. After the marathon, Coll2-1 and Coll2-1NO_2_ concentrations were slightly but systematically decreased. The total and active MPO concentrations were increased by 2 to 3-fold in comparison to the pre-marathon values (p < 0.001 for total and active MPO). The active MPO/total MPO ratio was significantly enhanced after the marathon (p < 0.001). The variation of total MPO during the marathon was negatively correlated with the training time per week (r = −0.34; p = 0.009).

The serum levels of Coll2-1 and Coll2-1NO_2_ were slightly decreased by marathon, indicating that intensive running could reduce cartilage catabolism. Furthermore, Coll2-1NO_2_ was not correlated with the total and active MPO indicating that Coll2-1 nitration did not result of a systemic oxidative phenomenon but reflects local changes.

## Introduction

Intensive exercises such as marathon running, induce a systemic inflammatory syndrome characterized by neutrophilia and increased plasmatic concentration of myeloperoxidase (MPO), pro-inflammatory cytokines such as interleukin (IL)-1, -6, -8, tumor necrosis factor (TNF) alpha and granulocyte-colony stimulating factor (G-CSF) (Suzuki et al. 2003). Furthermore, they induce neutrophils and cytokines accumulation in damaged muscles (Fielding et al. 1993; Hellsten et al. 1997; Suzuki et al. 2000; Nieman 2000) and subsequently the release of myocellular proteins such as creatine kinase (CK) and myoglobulin into the circulation (Bruunsgaard et al. 1997; Suzuki et al. 1999; Suzuki et al. 2000). It has further been hypothesized that marathon could increase cartilage matrix turnover, a phenomenon suspected to be related to systemic inflammatory syndrome. This hypothesis is based on the observation that the increased serum level of cartilage oligomeric matrix protein (COMP) during marathon is associated with the early release of soluble IL-6 receptor (sIL-6R), TNFα, IL-1 receptor antagonist (IL-1Ra) and soluble TNF receptors II (sTNFRII) (Camus et al. 1997; Northoff and Berg 1991; Nehlsen-Cannarella et al. 1997; Ostrowski et al. 1998; Neidhart et al. 2000). However, there is no evidence whether the serum level of COMP reflects pathological changes occurring in cartilage. Indeed, COMP is present in cartilage but also in other tissues such as tendons and synovial membrane (Neidhart et al. 1997; Di Cesare et al. 1997; Hummel et al. 1998 which might also be activated during or after physical exercises. In addition, COMP is released into the synovial fluid and blood in its intact or fragmented forms. This matter possibly reflects either the simple turnover or cartilage breakdown. In general, the immunoassay used in these studies is not able to differentiate the various fragmented forms of COMP. Consequently, it cannot be concluded if the increased serum levels represent either cartilage catabolism or just an elevated metabolic rate. On the other hand, Sweet *et al.* (Sweet et al. 1992) have shown that marathon race did not increase the serum levels of other markers such as keratan sulfate from the departure to 48 hours after the completion of the race. Therefore, the effects of long distance running on cartilage metabolism remain questionable. Moreover, several studies investigating cartilage by magnetic resonance imaging (MRI) have shown that competitive or leisure long-distance runners experienced no major short-term or long-term knee cartilage damages, considering that there was no evidence of pre-existing damage (Lazzarini et al. 1997; Colbert et al. 2000; Lohman et al. 2001; Krampla et al. 2008; Schueller-Weidekamm et al. 2006).

To investigate the effects of long-distance running on type II collagen metabolism, we have measured Coll2-1 and Coll2-1NO_2_ in the serum of marathon runners before and immediately after the race. Coll2-1 is a nine amino acid sequence (^108^HRGYPGLDG^116^ ) located in the helical part of type II collagen molecule, a specific and major protein of articular cartilage. Coll2-1 is a well-recognized marker of type II collagen catabolism (Deberg et al. 2005a; Huebner et al. 2010). Furthermore, this peptide contains a tyrosine which is sensitive to nitration. The nitrated form of Coll2-1 is called Coll2-1NO_2_ [^108^HRGY(NO_2_)PGLDG^116^] and represents the oxidation related to the cartilage degradation. Even if we cannot exclude that nitration occurs outside the joint, Coll2-1NO_2_ illustrates the oxidative-related cartilage matrix degradation. Recently, we have developed specific immunoassays (ELISA) to measure in serum Coll2-1 in its native or nitrated forms. The mean serum levels of these epitopes in adults aged 20–65 years did not vary with age and do not show diurnal variation. Coll2-1 and Coll 2-1NO_2_ were significantly elevated in osteoarthritic patients compared to age-matched controls (Henrotin et al. 2004). Additionally, the ratio Coll2-1NO_2_/Coll2-1 was shown to differentiate rheumatoid arthritis (RA) from osteoarthritis (OA) with a 1.6 fold higher ratio in RA than in OA (Deberg et al. 2005a). Finally, high levels of Coll2-1 and Coll2-1NO_2_ in the urine of OA patients have been shown to predict the progression of radiographic joint space narrowing over one year (Deberg et al. 2005b).

In this article, we report Coll2-1 and Coll2-1NO_2_ serum levels and the levels of other biochemical markers (MPO and C-reactive protein (CRP)) to further elucidate the effects of marathon running on cartilage degradation. MPO is a hemoprotein expressed in the primary granules of neutrophil polymorphonuclear leukocytes which uses hydrogen peroxide (H_2_O_2_) and Cl^-^ to form a powerful oxidant, hypochlorous acid (HOCl) (Deby-Dupont et al. 1999). MPO also catalyzes tyrosine nitration in proteins from nitrite and H_2_O_2_ (Sampson et al. 1998). Therefore, we hypothesize that MPO could be responsible for Coll2-1 peptide nitration and that Coll2-1 nitration could occur outside the cartilage. Indeed, MPO is not expressed in cartilage. To verify this hypothesis, we have measured Coll2-1NO_2_ in the serum of marathon runners just after the race. At this time, it is known that a high concentration of MPO is released in the blood and a systemic oxidative stress occurs (Camus et al. 1997; Melanson et al. 2006). The absence of Coll2-1NO_2_ increase after the marathon would suggest that Coll2-1 nitration is not a systemic phenomenon. This study was designed: (1) to investigate the effects of long distance running on type II collagen catabolism; (2) to study the impact of a systemic oxidative stress on Coll2-1 peptide nitration; (3) to determine the change of the active fraction of MPO during marathon.

## Methods

### Population

The study was conducted in 98 participants (78 men and 20 women, mean age (SD): 45.84 (7.77) years; range: 27.00–60.00) at a 42.195 Km marathon. Their demographic characteristics are summarized in Table 1. All were non-smokers, in good health with no history of febrile disease in the month before the race. None were taking any medication within two weeks before the race. None were subjected to bone radiological and/or scintigraphy and none had any evidence of renal or liver failure, arthritis or other inflammatory diseases. None was currently taking any medication known to modify arthritic disease or influence joint metabolism.

**Table 1:** **Anthropometric data, training history and performance of marathon runners (n = 98)**

Variables	Mean (SD)	Range
Age (year)	45.84 (7.77)	27–60
Sex ratio (Male/Female; %)	79.6/20.4	–
BMI (kg/m^2^)	22.9 (2.00)	18.37–27.76
Weekly training (min)	371.05 (207.67)	120.00–900.00
Performance [race time (min)]	195.56 (33.35)	94.00–304.00

The runners completed the race in an average of 198.81 ± 30.94 minutes (mean ± SD; ranged from 148 to 304 min). All the runners were deemed in medically stable condition at the end of the marathon. The study was approved by the local ethics committee. Subjects were informed of the experimental procedures and possible risks, and signed a letter of informed consent before participating.

### Blood sampling

The blood samples were collected at rest before and within 30 min after the race. Peripheral blood samples were collected in Vacutainers by antecubital venipuncture with the subject in a sitting position. Samples were allowed to clot 30 min at room temperature before being centrifuged at 1000 g for 10 min. The sera were aliquoted and stored at −80°C until their analysis. All samples were thawed only once.

### Immunoassays for Coll2-1 and Coll2-1NO_2_

Coll2-1 and Coll2-1NO_2_ concentrations were measured by two competitive and specific immunoassays (ELISA) (Deberg et al. 2005a). The Coll2-1 immunoassay measured the amino acid sequence ^108^HRGYPGLDG^116^ in its linear form whereas the Coll2-1NO_2_ immunoassay quantified the nitrated amino acids sequence. The limits of detection were 17 nM for Coll2-1 immunoassay and 25 pM for Coll2-1NO_2_ immunoassay. The intra- and inter-assays coefficients of variation (CV) were lower than 10% and the dilution curves were parallel to the standard curve for both assays. The analytical recoveries were in mean 104.7% and 121.9% for Coll2-1 and Coll2-1NO_2_ assays respectively.

Briefly, microplates were coated with 200 μl of streptavidine 0.5 μg/ml during 48 hours. After washing (washing buffer: Tris 25 mM, NaCl 50 mM, Tween 20 0,2% (v/v) pH 7.3), microtiter plates (Maxisorp, Nunc, Denmark) were blocked with 400 μl/well of blocking buffer (KH_2_PO_4_ 1.5 mM, Na_2_HPO_4_ 8 mM, KCl 2 mM, NaCl 138 mM, BSA 0.5% (v/v) pH 7.2) overnight at 4°C. Coll2-1 and Coll2-1NO_2_ peptides were conjugated to biotin according to the method described by Rosenquist *et al.* (Rosenquist et al. 1998). One hundred μl of the biotinylated peptides (Coll2-1 at 2.5 ng/ml or Coll2-1NO_2_ at 1.25 ng/ml) were added to each well of the streptavidine-coated plates and incubated for two hours at room temperature. After washing, 50 μl of calibrators (synthetic peptide) or unknown samples, diluted in incubation buffer (10 mM phosphate buffer saline (PBS), 138 mM NaCl, 7% (w/v) BSA, 0.1% (v/v) Tween 20 pH 7.0 for the Coll2-1 immunoassay and in 50 mM Tris, 138 mM NaCl, 7% (w/v) BSA, 0.1% (v/v) Tween 20 pH 8.0 for the Coll2-1NO_2_ immunoassay), were added to the wells, followed by either 100 μl of D3 antibody (for Coll2-1, diluted 1/40,000) or 100 μl of D37 antibody (for Coll2-1NO_2_, diluted 1/500,000) and incubated one hour at room temperature. The dilutions of the antisera and of the secondary antibody were done in dilution buffer (10 mM PBS, 138 mM NaCl, 0.2% (w/v) BSA, 0.1% (v/v) Tween 20 pH 7.0 for the Coll2-1 immunoassay and in 50 mM Tris, 138 mM NaCl, 0.2% (v/v) BSA, 0.1% (v/v) Tween 20 pH 8.0 for the Coll 2-1NO_2_ immunoassay). One hundred μl of peroxidase-conjugated goat antibodies to rabbit IgG (Biosource, Belgium), diluted 1/5000 in incubation buffer, were incubated one hour at room temperature. Washing steps were performed between each incubation. Finally, 100 μl of freshly prepared enzyme substrate [3,3’,5,5’-Tetramethylbenzidine (TMB), Biosource, Belgium] were added into each well. After 15 minutes, the reaction was stopped with 100 μl of 4M H_3_PO_4_. The coloration was read with a microplate reader (Labsystem, Finland) at 450 nm, corrected for absorbance at 650 nm.

### Total MPO by ELISA

The total MPO concentration in sera was measured by a solid phase two-site enzyme linked immunosorbent assay, as described by the manufacturer (ELIZEN MPO, Zentech SA, Liège, Belgium), in which the capture and the detection antibodies were two polyclonal antibodies. The performances of the assay were: a sensitivity equal to 0.4 ng/ml, the inter- and intra-assays CV lower than 15%, an accuracy between 89 and 104% and the dilution curves parallel to the standard curve.

### Active MPO by SIEFED (Specific immunological extraction followed by enzymatic detection)

. The measurement of active MPO was realized as described by Franck *et al* (Franck et al. 2006) and adapted for human MPO activity measurement (Franck et al. 2009). Briefly, MPO in the biological fluid was captured on specific immobilized antibodies. After washing to eliminate unspecifically bound compounds or interfering substances, the detection of MPO enzymatic activity was performed by using H_2_O_2_ as substrate, Amplex Red as fluorogenic electron donor and nitrite as enhancer of the reaction. The intra- and inter-assay CV were under 10 and 20%, respectively. The limit of detection was 0.40 ng/ml.

### Biochemical measurements standard

Serum samples pre-and post-marathon were analyzed on the Roche/Cobas Integra 400/700/800 system (Roche Diagnostics) using reagents supplied by the manufacturer. Total proteins, albumin and uric acid in sera were measured spectrophotometrically. Total creatine kinase (CK) was determined by a kinetic method. CRP (C-reactive protein) was measured by immunoturbimetry (Roche Diagnostcis GmbH, Mannheim, Germany).

### Statistical analysis

The Coll2-1, Coll2-1NO_2_, CK, uric acid, albumin, total protein, CRP, total MPO, active MPO and active MPO/total MPO ratio values measured before and after the marathon race were expressed as median (range). Statistical analyses were carried out with GraphPad InStat3. Wilcoxon matched pairs test was used to compare each biological marker values before and after the marathon race. To determine the influence of gender, age, BMI on biological markers (Coll2-1, Coll2-1NO_2_, total MPO, active MPO, CRP, albumin, total protein, uric acid, creatine kinase) and on marathon characteristics (training time and performance), a regression analysis was performed. The correlations between the different biological markers before and the variation during the marathon were estimated by the non-parametric Spearman’s rank correlation coefficient. Data were considered statistically significant when P value was below 0.05 (two-tailed test).

## Results

### Cartilage biomarkers

The levels of Coll2-1 and Coll2-1NO_2_ measured right before the race were not affected by age, BMI, sex, training time and performance. Coll2-1 and Coll2-1NO_2_ concentrations were significantly decreased after the marathon, [Coll2-1 pre: 107.79 (28.77–296.94) nM; Coll2-1 post: 93.45 nM (28.89–278.87) (p < 0.001) and Coll2-1NO_2_ pre: 0.26 (0.05–0.71) nM, Coll2-1NO_2_ post: 0.21 (0.05–0.61) (p < 0.001)] (Table 2). The reduction was observed in 73.7% of the participants for Coll2-1 and in 57.9% of participants Coll2-1NO_2_. There was no difference between men and women values before and after the race.

**Table 2:** **Pre- and post-marathon values of biochemical markers**

Marker	Pre-marathon	Post-marathon	Significance
Total protein (mg/ml)	78.21 (67.54–90.55)	81.40 (65.16–92.30)	<0.001
Albumin (g/l)	45.00 (40.00–53.00)	47.00 (41.00–54.00)	<0.001
Uric Acid (mg/l)	47.00 (25.00–72.00)	55.00 (31.00–80.00)	<0.001
Creatine kinase (U/l)	59.00 (11.00–200.00)	212.00 (44.00–2020.00)	<0.001
CRP (mg/l)	0.84 (0.00–8.00)	1.06 (0.00–12.00)	<0.001
Total MPO (ng/ml)	42.30 (<0.40–343.39)	94.40 (13.47–465.30)	<0.001
Active MPO (ng/ml)	4.05 (<0.40–50.68)	11.63 (<0.40–175.23)	<0.001
Active MPO/Total MPO ratio (%)	7.73 (0.00–61.49)	13.72 (0.07–92.68)	<0.001
Coll2-1 (nM)	107.79 (28.77–296.94)	93.45 (28.89–278.87)	<0.001
Coll2-1NO_2_ (nM)	0.26 (0.05–0.71)	0.21 (0.05–0.61)	<0.001

### Neutrophil activation and systemic inflammation markers

The pre-marathon levels of total MPO measured right before the race were significantly higher in female [median: 57.70 (<0.40–216.74) ng/ml] than in male [median: 38.40 (15.60–343.39) ng/ml] (p < 0.05). By contrast, the levels of CRP were not statistically different [male 0.84 (0.00–7.00) mg/l and female 0.83 (0.00–8.00) mg/l]. The serum levels of CRP and total MPO were not correlated with age, BMI, performance and training time before the marathon. After the marathon, the levels of MPO and CRP increased in 89% and 15.4% of the participants respectively [MPO pre: 42.30 (<0.40–343.49) ng/ml and MPO post: 94.40 (13.47–465.30) ng/ml; CRP pre: 0.84 (0.00–8.00) mg/l and CRP post: 1.06 (0.00–12.00) mg/l] (Table 2). No difference was observed between men and women after the race.

Before the marathon, there was no difference of active MPO between men and women [men: 4.15 (<0.40–50.68) ng/ml and women 3.46 (<0.40–33.35) ng/ml]. No correlation between pre-marathon active MPO values and BMI, age, training time and performance was observed. After the marathon, the active MPO was significantly increased [pre-marathon values: 4.05 (<0.40–50.68) ng/ml and post-marathon values: 11.63 (<0.40–175.23) ng/ml (p < 0.0001)]. After the marathon, no difference between men and women was observed [men: 11.73 (0.86–175.23) ng/ml and women 7.83 (<0.40–28.73) ng/ml]. Finally, in comparison with the pre-marathon values, the ratio active MPO/total MPO was increased after the race [pre-marathon: 7.73 (0.00–61.49) % vs post-marathon: 13.72 (0.07–92.68) % (p < 0.0001)]. A positive correlation was found between active MPO and total MPO (r = 0.67; p < 0.001). No relationship was observed between MPO activity and CRP, Coll2-1NO_2_.

### Biochemical markers

Before the marathon, total protein and albumin values were not affected by age, BMI, sex, training time and performance at the marathon. After the marathon, total protein and albumin concentrations significantly increased [total protein pre-marathon: 78.21 (67.54–90.55) mg/ml; post-marathon: 81.40 (65.16–92.30) mg/ml and albumin pre-marathon: 45 (40.00–53.00) g/l; post-marathon: 47.00 (41.00–54.00) g/l; p < 0.001]. The elevations of total protein and albumin levels were moderate (total protein: 3.64 ± 0.45% and albumin: 3.88 ± 0.44%) but were present in all subjects.

Pre-marathon CK levels were more elevated in male [median: 62.00 (22.00–200.00) U/l/] than in female [median: 54.50 (11.00–164.00) U/l] but the difference was not significant (p = 0.074). No correlation of pre-marathon CK values and age, BMI, training and performance was observed. After the race, CK significantly increased by an average of 3.6 fold [pre-marathon: 59.00 (11.00–200.00) U/l; post-marathon: 212.00 (44.00–2020.00) U/l] (Table 2). The increase of CK levels and the post-marathon levels were similar in men and women.

Pre-marathon serum uric acid levels were more elevated in men [48.00 (32.00–72.00) mg/l] than in women [36.00 (25.00–67.00) mg/l] (p < 0.001). No correlation between pre-marathon uric acid values and the BMI, the training time and the performance was observed. After the marathon, the uric acid concentrations were increased by 1.17-fold [pre: 47.00 (25.00–72.00) mg/l; post: 55.00 (31.00–80.00) mg/l] (p < 0.001) (Table 2) and the difference between men and women levels was still present [men: 57.00 (35.00–77.00) mg/l and women: 44.00 (31.00–80.00) mg/l] (p = 0.01).

### Correlations between biological markers, demographic characteristics before the race and changes in serum concentrations after the marathon

**Figure 1:**
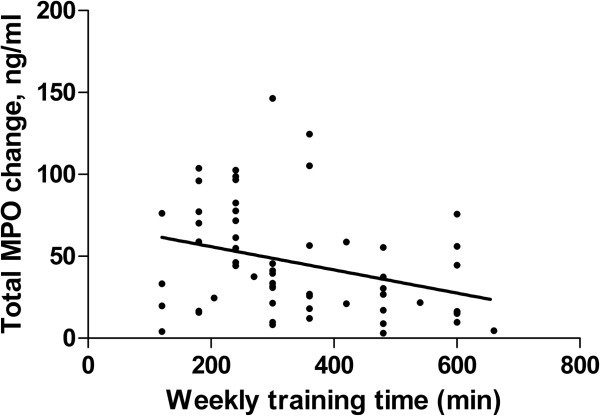
**Correlation (r = −0.34, p = 0.009) between the variation of total MPO during the marathon and the time of weekly training.**

A multiple regression analysis was employed to test to what extent the variability of changes in biological markers during the marathon could be explained by demographic parameters, training time or performance. A significant positive correlation was found for total protein changes during marathon and BMI (r = 0.24, p = 0.02) as well as for albumin changes and age (r = 0.21; p = 0.05). The variation of total MPO levels during the race, but not active MPO or active MPO/total MPO ratio, was negatively correlated with the weekly training time (r = −0.34, p = 0.009) (Figure 1). The changes in active MPO levels during the marathon was negatively correlated with the age (r = −0.25, p = 0.03). The other biomarkers changes were not correlated with age, BMI, training time or performance.

None of the biomarkers levels before the race was correlated with changes during the marathon.

## Discussion

Previous studies, that investigated acute stress on the knee caused by marathon running, revealed controversial results most likely due to their small number of participants and the crossed distance (Neidhart et al. 2000; Krampla et al. 2001; Wu et al. 2004; Schmitt et al. 2006; Schueller-Weidekamm et al. 2006; Kim et al. 2007).

The main result of our study was that the serum levels of Coll2-1 and Coll2-1NO_2_ slightly, but systematically and significantly, decreased after the marathon, whereas total protein concentrations increased. This finding indicates that the changes in Coll2-1 and Coll2-1NO_2_ levels do not result of a global change in connective tissue metabolism, but well of a change in cartilage metabolism. To our knowledge, this study is the first one to demonstrate the effects of an intensive running on collagen catabolism. Previous studies have reported that the serum levels of keratan sulfate, a component of aggrecan, were unchanged in runners immediately after a marathon (Sweet et al. 1992), and that the serum levels of COMP were increased during and after the marathon (Neidhart et al. 2000). In human, COMP is produced in cartilage by chondrocytes and in tendons, menisci, and synovial tissue by fibroblasts (Di Cesare et al. 1997; Hummel et al. 1998). Therefore, the rise in COMP serum concentrations during the marathon may be indicative of the severe physical strain on joint structure, but may also be associated with tendonitis, synovitis or meniscus injury. In contrast, Coll2-1 is specific of cartilage tissue. Coll2-1 is a denaturation epitope located in triple helical domain of the type II collagen molecule that is made available by unwinding of the triple helix (Henrotin et al. 2007). Coll2-1 and Coll2-1NO_2_ were found to be elevated in the serum of OA patients (Deberg et al. 2005a) and in OA cartilage (Deberg et al. 2008. These observations revealed that Coll2-1 was a specific marker of the cartilage degradation. Therefore, the decrease in Coll2-1 levels in post-marathon serum could be interpreted as a protective effect of long distance running on cartilage. This hypothesis is supported by the works of Neidhart *et al.* (Neidhart et al. 2000) that demonstrated that the serum levels of IL-1 receptor antagonist (IL-1Ra) highly increased during the marathon and remained elevated two hours after the marathon completion, whereas the serum levels of IL-1β were unchanged. IL-1Ra competes with IL-1 for the same receptor without having intrinsic activity. By this way, IL-1Ra limits the deleterious effect of IL-1β on cartilage (Caron et al. 1996). IL-1β is known to be the key cytokine in cartilage degradation in OA. IL-1β stimulates the production of matrix metalloproteases and reactive oxygen species by chondrocytes and decreases the synthesis of cartilage matrix constituents. Therefore, we could conclude that the decrease of the serum levels of serum Coll2-1 at the completion of the marathon, could result of the inhibition of IL-1β activity by IL-Ra.

Another explanation could be that the decrease in the serum level of Coll2-1 results of an increase in Coll2-1 clearance secondary to prolonged exercise as previously observed with MPO in marathon (Suzuki et al. 2003). As previously reported, the serum levels of MPO were highly increased after marathon indicating that long distance race induces neutrophil degranulation (Camus et al. 1997). For the first time, we have also measured serum active MPO using an original method called SIEFED (‘Specific Immunological Extraction Followed by Enzymatic Detection’) allowing the study of the enzyme without interference of the biological sample. In this assay, first, blood MPO was captured on specific immobilized antibodies, secondly, not specifically bound compounds or interfering substances were eliminated by washings and, third, MPO enzymatic activity was revealed fluorimetrically. With this method, we have found that not only total MPO, but also active MPO were increased suggesting that a systemic oxidative stress and a production of hypochloric acid and derivatives oxidants could occur after a marathon. Interestingly, MPO and active MPO before or after the marathon were not correlated with Coll2-1NO_2_. This finding supports the concept that Coll2-1 nitration does not result of systematic oxidative stress, but rather reflects oxidative stress occurring in the joints. The variation of total MPO during the marathon, but not active MPO or active MPO/total MPO ratio, was negatively correlated with the weekly training time. This result indicates that the training prevents from neutrophils degranulation during marathon. This finding also suggests that the blood concentration of total MPO could be a useful marker for the follow-up of training.

Another important observation was that a fraction of the released MPO is activated during the marathon. The *in vivo* regulation of MPO is complex. Indeed the enzyme performs its function in a wide variety of environments with different pH and levels of NO, H_2_O_2_, O_2_^.-^, O_2_, inorganic and organic substrates, and reducing agents (Klebanoff 2005). Two hypotheses can be proposed to explain the increased levels of active MPO after the race. First, it could be due to the decrease of the serum levels of lipoprotein, an inhibitor of active MPO, immediately and until one day after a marathon (Goodyear et al. 1990). Secondly, the peroxidase activity of MPO could be down regulated by NO (Abu-Soud and Hazen 2000). As a significant reduction in the excretion of NO metabolites has been demonstrated in marathon runners, we can speculate that the decrease of NO metabolites in serum promote MPO activity (Rodriguez-Plaza et al. 1997). Although we observed for total MPO a difference between men and women before the marathon race, it is difficult to assert that this difference is only due to gender. Indeed, many factors such as smoking, oral contraception, anthropomorphic variables, can influence the concentration of MPO (Hoy et al. 2001). However an important observation was that a fraction of the released MPO is active during the marathon whatever the gender. The *in vivo* regulation of MPO is complex. The idea was already explained above.

## Conclusions

Our data reveal 1) that long distance running slightly but significantly decreases type II collagen catabolism. This finding suggests that long-distance running does not damage cartilage at short-term; and 2) that the active fraction of MPO increases during stressful running race.
